# Cytochrome P450 2D6 profiles and anti-relapse efficacy of tafenoquine against *Plasmodium vivax* in Australian Defence Force personnel

**DOI:** 10.1128/aac.01014-23

**Published:** 2023-11-16

**Authors:** Simone Dowd, Nanhua Chen, Michelle L. Gatton, Michael D. Edstein, Qin Cheng

**Affiliations:** 1 Australian Defence Force Malaria and Infectious Disease Institute, Brisbane, Australia; 2 Centre for Immunology and Infection Control, Faculty of Health, Queensland University of Technology, Brisbane, Australia; The Children's Hospital of Philadelphia, Philadelphia, Pennsylvania, USA

**Keywords:** *Plasmodium vivax*, relapse, tafenoquine, CYP2D6, metabolism

## Abstract

*Plasmodium vivax* infections and relapses remain a major health problem for malaria-endemic countries, deployed military personnel, and travelers. Presumptive anti-relapse therapy and radical cure using the 8-aminoquinoline drugs primaquine and tafenoquine are necessary to prevent relapses. Although it has been demonstrated that the efficacy of primaquine is associated with Cytochrome P450 2D6 (CYP2D6) activity, there is insufficient data on the role of CYP2D6 in the anti-relapse efficacy of tafenoquine. We investigated the relationship between CYP2D6 activity status and tafenoquine efficacy in preventing *P. vivax* relapses retrospectively using plasma samples collected from Australian Defence Force personnel deployed to Papua New Guinea and Timor-Leste who participated in clinical trials of tafenoquine during 1999–2001. The CYP2D6 gene was amplified from plasma samples and fully sequenced from 92 participant samples, comprised of relapse (*n* = 31) and non-relapse (*n* = 61) samples, revealing 14 different alleles. CYP2D6 phenotypes deduced from combinations of CYP2D6 alleles predicted that among 92 participants 67, 15, and 10 were normal, intermediate, and poor metabolizers, respectively. The deduced CYP2D6 phenotype did not correlate with the corresponding participant’s plasma tafenoquine concentrations that were determined in the early 2000s by high-performance liquid chromatography or liquid chromatography-mass spectrometry. Furthermore, the deduced CYP2D6 phenotype did not associate with *P. vivax* relapse outcomes. Our results indicate that CYP2D6 does not affect plasma tafenoquine concentrations and the efficacy of tafenoquine in preventing *P. vivax* relapses in the assessed Australian Defence Force personnel.

## INTRODUCTION

*Plasmodium vivax* is responsible for almost half of all malaria cases outside of Africa (1) and is the most geographically widespread human *Plasmodium* species (2). While it is predominantly prevalent in the Asia Pacific, Central and South America, and the Horn of Africa region, *P. vivax* is present across almost all malaria-endemic regions of Africa (3). Although it was historically thought to be benign, *P. vivax* infections have been increasingly shown to cause enormous disease and economic burdens in endemic countries (4, 5).

It is difficult to cure *P. vivax* due to its ability to form hypnozoites in the host liver following an inoculation of sporozoites from an infected mosquito. This dormant parasite stage activates to cause relapses weeks to months after the initial infection. Tropical *P. vivax* usually relapses 3 weeks after the initial infection whereas infections in temporal areas show a long latency phenotype, with relapses occurring at 8 to 10 months after the initial infection (6). Studies in Papua New Guinea (PNG) estimated that 70% to 82% of *P. vivax* infections in children were a result of relapses (7, 8). A separate study estimated that relapse was responsible for approximately 96% of *P. vivax* infections in endemic areas of Thailand in children less than 15 years of age (9). Overall, relapses account for ≥79% of recurring *P. vivax* (10). Multiple episodes of *P. vivax* infections have been shown to increase the risk of hospitalization and death (5). The frequency and burden of relapses highlight the importance of eliminating hypnozoites from an individual’s liver when treating *P. vivax* infections.

Currently, the WHO recommends “radical cure” treatment using a 3-day course of a blood schizontocidal drug plus a 14-day course of primaquine (PQ) to target the hypnozoite reservoir in the liver and prevent relapses and further transmission (11). The 8-aminoquinoline drugs, which include PQ and tafenoquine (TQ), are the only class of drugs found effective against both *P. vivax* hypnozoites to achieve radical cure and *P. falciparum* gametocytes to reduce transmission (11, 12). However, individual adherence to the 14-day PQ regimen is less than ideal, reducing the effectiveness of radical cure (13, 14).

In 2018, the United States Food and Drug Administration and the Australian Therapeutic Goods Administration approved the use of TQ, in combination with a course of schizontocidal drug, as a radical cure for *P. vivax* and for malaria prophylaxis. TQ offers a promising alternative to PQ for radical cure as it has an elimination half-life of approximately 2 weeks (15) compared to PQ’s half-life of approximately 4–6 hours (16, 17), effectively eliminating the requirement for daily drug dosing.

TQ can also be taken weekly for up to 6 months as a chemoprophylaxis. Currently, it is not recommended in children (<18 years of age) and pregnant women. As with PQ, TQ is not used in individuals with glucose-6-phosphate dehydrogenase (G6PD) deficiency, a hereditary X-linked condition responsible for drug-induced hemolysis, the severity of which is directly linked to the individual’s level of G6PD enzyme activity and the dose of the drug (18, 19).

The human cytochrome P450 2D6 (CYP2D6) gene codes for the cytochrome P450 hepatic enzyme, which is responsible for the metabolism of 20%–30% of all clinical pharmaceuticals currently available (20, 21). Polymorphisms identified in the CYP2D6 gene result in variations in the level of metabolism of drugs reliant upon this pathway. To date, over 160 allelic variants have been documented, which are separated into four categories: increased function (IF), fully functional (FF), reduced function (RF), and nonfunctional (NF). The combination of these allelic variants for an individual determines their CYP2D6 metabolic activity phenotype: ultrarapid metabolizer (UM), normal metabolizer (NM), intermediate metabolizer (IM), and poor metabolizer (PM) (22).

PQ metabolism in humans utilizes two pathways for metabolite conversion: the MAO-A pathway converting PQ to inactive carboxyprimaquine and the CYP2D6 pathway where hydroxylation generates phenolic metabolites with redox activity. The latter is believed to be responsible for the hypnozoiticide efficacy and hemolytic activity of PQ when there is a deficiency in the G6PD enzyme (23). There has been substantial evidence that CYP2D6 phenotype or genotype-predicted activity score is a significant factor in PQ metabolism and an IM or PM is at increased risk of PQ failure with *P. vivax* relapse (24–26).

In murine models, CYP2D gene knockout completely blocked the anti-liver stage activity of PQ, and this activity could be partially restored using humanized CYP2D6 knock-in mice (27). Like PQ, mouse models have shown that TQ’s activity against liver-stage parasites was suppressed in CYP2D6 knocked-out mice, but partial activity was restored in humanized CYP2D6 knock-in mice although requiring a higher TQ dose (28). CYP2D6 knockout also affected the blood TQ exposure, though with a much less reduction compared to PQ (29). These studies suggest that the CYP2D6 gene may affect TQ pharmacokinetics and anti-liver stage activity, though to a lesser extent than PQ.

Two clinical studies have been conducted to investigate the influence of CYP2D6 activity on the anti-relapse efficacy of TQ (30, 31). The first study showed that an individual’s CYP2D6 metabolism status was not associated with TQ anti-relapse efficacy (30). The second study demonstrated that the frequency of recurrent *P. vivax* infections following TQ radical cure was not associated with participants’ CYP2D6 activity scores (AS) (31). As the number of participants with CYP2D6-PM status in the TQ arm was zero in the first study and three in the second study, the impact of the human CYP2D6-PM phenotype on the anti-relapse efficacy of TQ remains unanswered.

The aim of this study was to determine whether participants’ CYP2D6 activity status influences plasma TQ concentrations and the anti-relapse efficacy of TQ. We examined historical plasma samples collected from deployed Australian military personnel treated with TQ to genotype their CYP2D6 status and predict CYP2D6 activities. We then examined if previously determined plasma TQ concentrations and TQ anti-relapse efficacy were reliant on the deduced CYP2D6 phenotype (genotype predicted CYP2D6 activity score and metabolic status). Our results suggest that unlike PQ, TQ concentrations and anti-relapse efficacy are likely not dependent upon the individual’s CYP2D6 activity status.

## RESULTS

**Fig 1:**
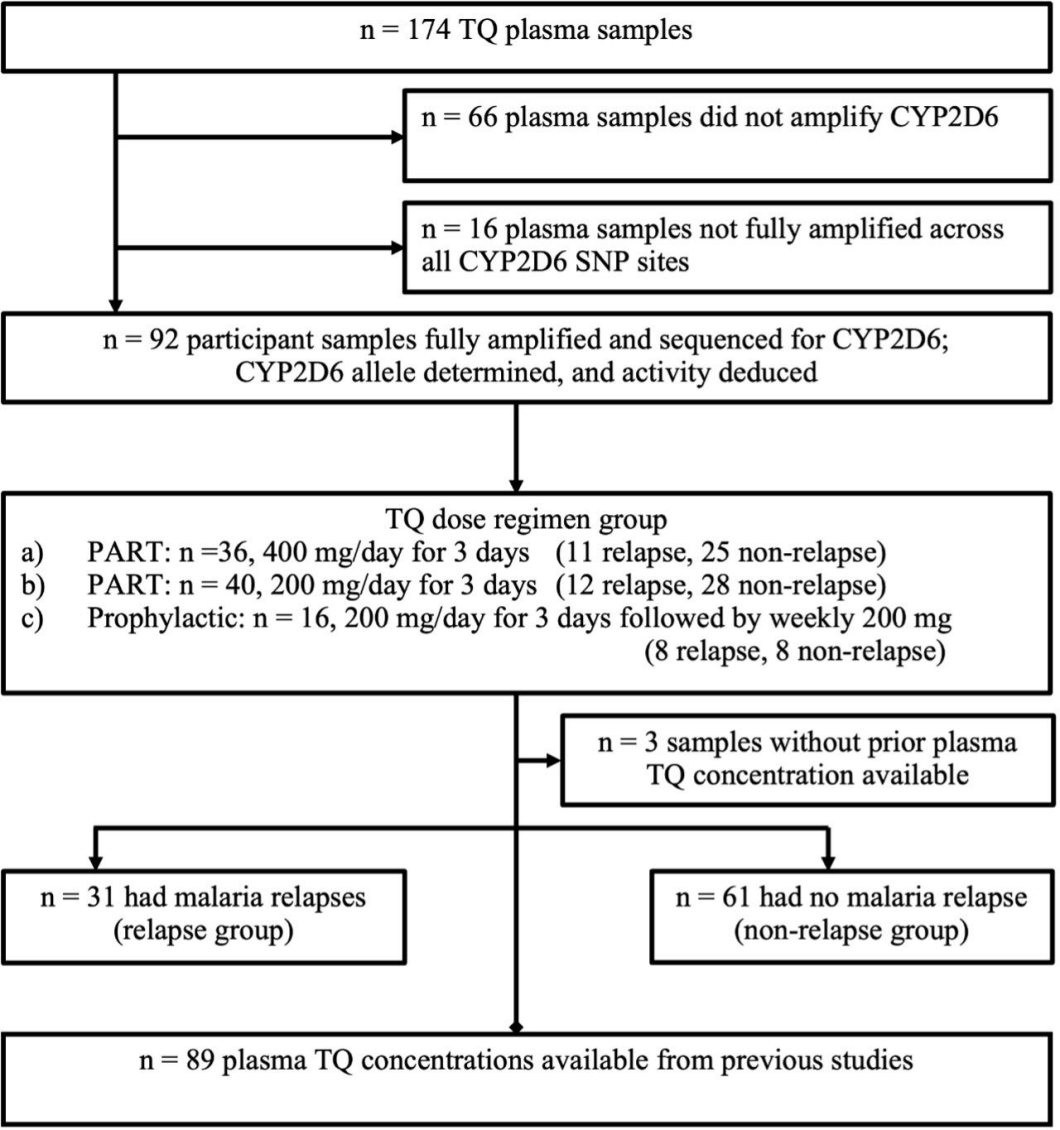
Flowchart illustrating the study design, number of participants, and plasma samples collected from the participants.

This was a retrospective study using human plasma samples collected as part of clinical trials of TQ conducted between 1999 and 2001. Plasma samples from the participants who had experienced a *P. vivax* relapse (relapse group) and from a randomly selected subset of participants who did not experience a *P. vivax* infection (non-relapse group) after TQ treatment were used to amplify the full-length CYP2D6 gene and sequenced. Of the 174 plasma samples tested, 92 (53%) were successfully amplified to complete the CYP2D6 gene sequence to identify all single nucleotide polymorphisms of interest. Of these 92 samples, 89 (30 relapse and 59 non-relapse) had plasma TQ concentration data available from the earlier studies (32, 33) (Fig. 1). The three samples (one relapse, two non-relapse) that did not have plasma TQ concentrations were used only for CYP2D6 profile and statistical analysis, which did not require TQ concentrations.

### CYP2D6 alleles and allele frequency

CYP2D6 sequence analysis of 92 samples revealed 174 alleles, of which 14 different alleles were identified: 11 each in the relapse and non-relapse groups (see Table S1). The predominant allele for both groups was *1, the wild-type fully functioning allele followed by *4, a nonfunctional allele. Overall, the wild-type allele accounted for 44% of alleles in the complete set of samples, with fully functional alleles accounting for 60%. Three gene duplication alleles (*2XN, 1.7%) were detected, and one of the original 174 samples had a gene deletion (0.57%).

### Allele combinations and frequency

Table 1 displays the genotypes and frequencies observed within 92 participants. Among these participants, the wild-type genotype *1/*1 had the highest frequency (29.3%), followed by *1/*4 (9.8%) and *4/*4 (7.6%). The frequency of these major genotypes was not significantly different between the non-relapse and relapse groups (*P* > 0.05). The homologous and heterologous combination of alleles accounted for 59% and 41% of genotypes, respectively. Interestingly, individuals with a homologous combination of alleles had a significantly higher odds ratio of relapse (OR = 2.78, 95% CI: 1.08–7.18). The risk of relapse was even higher for individuals predicted to be IM, with a homologous allele combination (OR = 13.13, 95% CI: 1.92–89.52).

**TABLE 1:** CYP2D6 genotype and corresponding deduced metabolic status (MS), activity score (AS), and frequency in participant groups^*b*^

MS	Homo/ hetero	Genotype	AS	Non-relapse		Relapse		All		OR^*a*^	95% CI
				*n*	%	*n*	%	*n*	%		
UM	Homo	*2XN/*2XN	4	0	0.0%	1	3.2%	1	1.1%		
		*Subtotal*		*0*	*0.0%*	*1*	*3.2%*	*1*	*1.1%*		
	Subtotal			0		1	3.2%	1	1.1%	NA	NA
NM	Homo	*1/*1	2	19	31.1%	8	25.8%	27	29.3%		
		*2/*2	2	2	3.3%	1	3.2%	3	3.3%		
		*35/*35	2	1	1.6%	1	3.2%	2	2.2%		
		*Subtotal*		*22*	*36.1%*	*10*	*32.3%*	*32*	*34.8%*		
	Hetero	*2XN/*10	2.25	0	0.0%	1	3.2%	1	1.1%		
		*1/*35	2	2	3.3%	0	0.0%	2	2.2%		
		*1/*39	2	1	1.6%	0	0.0%	1	1.1%		
		*2/*1	2	3	4.9%	2	6.5%	5	5.4%		
		*2/*35	2	1	1.6%	0	0.0%	1	1.1%		
		*1/*41	1.5	1	1.6%	3	9.7%	4	4.3%		
		*2/*41	1.5	1	1.6%	0	0.0%	1	1.1%		
		*1/*10	1.25	3	4.9%	0	0.0%	3	3.3%		
		*2/*10	1.25	1	1.6%	0	0.0%	1	1.1%		
		*35/*10	1.25	2	3.3%	0	0.0%	2	2.2%		
		*Subtotal*		*15*	*24.6%*	*6*	*19.4%*	*21*	*22.8%*		
	Subtotal			37	60.7%	16	51.6%	53	57.6%	1.14	0.34–3.80
IM	Homo	*41/*41	1	3	4.9%	3	9.7%	6	6.5%		
		*9/*9	1	0	0.0%	3	9.7%	3	3.3%		
		*10/*10	0.5	1	1.6%	1	3.2%	2	2.2%		
		*Subtotal*		*4*	*6.6%*	*7*	*22.6%*	*11*	*12.0%*		
	Hetero	*1/*4	1	8	13.1%	1	3.2%	9	9.8%		
		*1/*5	1	1	1.6%	0	0.0%	1	1.1%		
		*1/*65	1	1	1.6%	1	3.2%	2	2.2%		
		*2/*3	1	1	1.6%	0	0.0%	1	1.1%		
		*2/*6	1	1	1.6%	0	0.0%	1	1.1%		
		*41/*4	0.5	3	4.9%	0	0.0%	3	3.3%		
		*Subtotal*		*15*	*24.6%*	*2*	*6.5%*	*17*	*18.5%*		
	Subtotal			19	31.1%	9	29.0%	28	30.4%	13.13	1.92–89.52
PM	Homo	*4/*4	0	4	6.6%	3	9.7%	7	7.6%		
		*5/*5	0	0	0.0%	1	3.2%	1	1.1%		
		*6/*6	0	1	1.6%	0	0.0%	1	1.1%		
		*7/*7	0	0	0.0%	1	3.2%	1	1.1%		
		*Subtotal*		*5*	*8.2%*	*5*	*16.1%*	*10*	*10.9%*		
	Subtotal			5	8.2%	5	16.1%	10	10.9%	NA	NA
Total	Homo			31	50.8%	23	74.2%	54	58.7%		
	Hetero			30	49.2%	8	25.8%	38	41.3%		
				61	66.3%	31	33.7%	92	100.0%	2.78	1.08–7.18

^*a*^ OR is the odds of relapse for homologous genotypes compared to heterologous genotypes.

^*b*^ UM, NM, IM, and PM represent ultrarapid, normal, intermediate, and poor metabolizers, respectively. Homo and hetero represent homologous and heterologous combinations, respectively.

### Predicted CYP2D6 phenotype

Each participant’s plasma sample had their CYP2D6 metabolism status classified based upon the allele combination as either UM, NM, IM, or PM and activity score (AS) calculated from 0 to 4. Among the 92 samples, 1, 67, 15, and 10 were classified as UM, NM, IM, and PM, respectively. The single UM sample was included in the NM group for statistical analysis. Fifty-four plasma samples had an AS >1 and 38 with an AS ≤1 (Table 1).

### Plasma TQ concentration versus TQ dosing regimen

**Fig 2:**
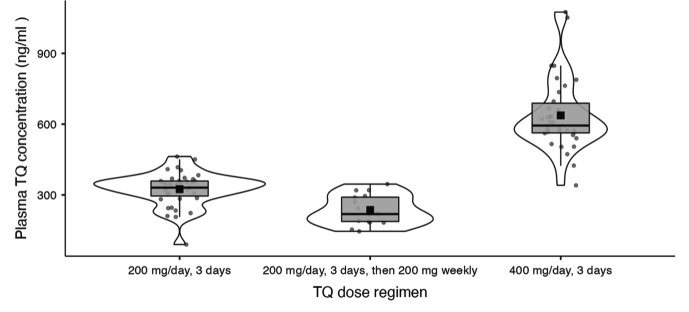
Box plot and violin plot of the distribution of plasma TQ concentrations in participants receiving three different TQ regimens. The boxes indicate the first and third quartiles, the median is indicated by the horizontal line, and the mean is indicated by a solid black square. The violin plot indicates the distribution.

**Fig 3:**
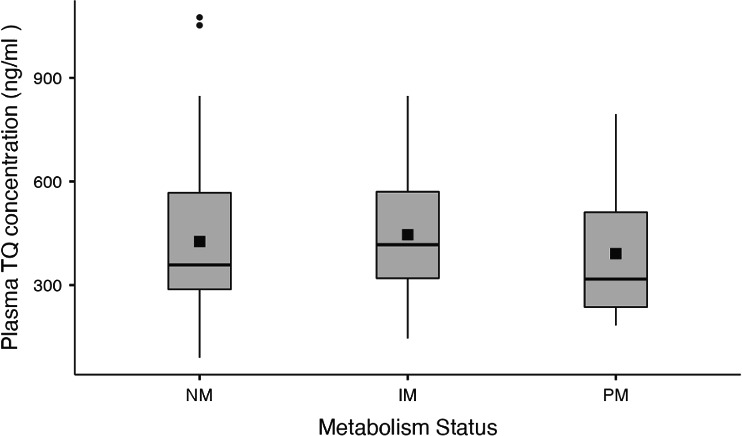
Plasma TQ concentrations in participants of different CYP2D6 metabolism statuses. The boxes indicate the first and third quartiles, the median is indicated by the horizontal line, and the mean is indicated by a solid black square.

Participant’s plasma TQ concentrations were compared between the three TQ dosing regimens. Pairwise comparisons revealed significant differences between all three regimens (*P* < 0.001). Individuals receiving 400 mg daily for 3 days had higher plasma TQ concentrations (mean 637 ± 156 ng/mL, *n* = 34) than those of individuals receiving 200 mg daily for 3 days (325 ± 71.5 ng/mL, *n* = 39), who had higher plasma TQ concentrations than those of individuals receiving a loading dose of 200 mg daily for 3 days followed by a weekly 200-mg dose (236 ± 63 ng/mL, *n* = 16) (Fig. 2). No association was found between CYP2D6 metabolism status (NM, IM, PM) and plasma TQ concentration (*P* = 0.493) (Fig. 3), suggesting that CYP2D6 does not affect the plasma concentrations of TQ.

### Relationship between plasma TQ concentration and relapse status

**Fig 4:**
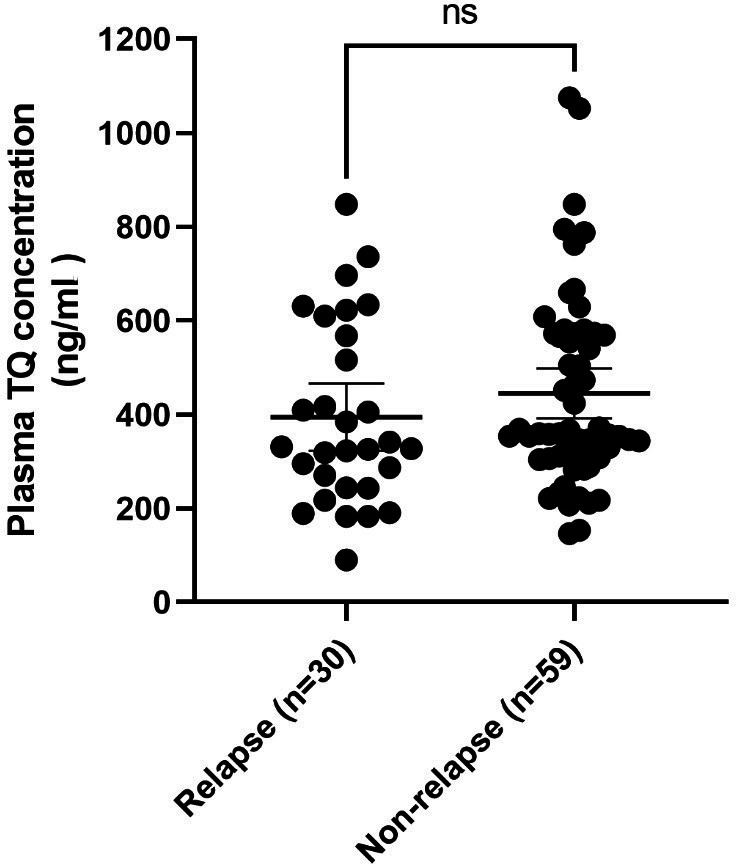
Comparison of plasma TQ concentrations in the relapse and non-relapse groups. The mean and 95% confidence intervals are represented as horizontal lines.

Plasma TQ concentrations were compared between participants in the relapse and non-relapse groups. The mean plasma TQ concentrations between participants in the relapse and non-relapse groups were 620 ± 135 ng/mL (*n* = 10) and 644 ± 166 ng/mL (*n* = 24), respectively, for the 400-mg TQ daily dose for 3 days. The corresponding values for the 200-mg TQ daily dose for 3 days were 322 ± 89.7 ng/mL (*n* = 12) and 326 ± 63.7 ng/mL (*n* = 27). The corresponding values for the loading dose of 200 mg TQ daily for 3 days followed by 200 mg TQ weekly were 221 ± 43.4 ng/mL (*n* = 8) and 252 ± 78.1 ng/mL (*n* = 8), respectively. No statistically significant difference was observed in participant’s plasma TQ concentrations between the relapse (395 ± 193 ng/mL, *n* = 30) and non-relapse (445 ± 204 ng/mL, *n* = 59) groups (*P* = 0.233, Fig. 4).

### Days to relapse and TQ dosing regimens

The number of days from the last TQ dose to relapse ranged from 67 to 354 days, with a mean of 158 days and a median of 139 days. There was no statistical significance between TQ dosing regimens and days to relapse (*P* = 0.496) (see Fig. S1 in the supplemental material).

### CYP2D6 metabolism status and relapse status

There was no significant difference in proportions of CYP2D6 NM, IM, and PM between the relapse and non-relapse groups (*P* = 0.512, Table 1). There was also no significant association between AS and relapse (*P* = 0.592). Although the non-relapse group had a higher proportion with AS >1 compared to that in the relapse group (60.7% versus 54.8%), the difference did not reach significance (*P* = 0.148) (Table 1). These results indicate that CYP2D6 metabolic activity status did not affect the relapse outcome among the participants treated with TQ.

### Relationship between CYP2D6 metabolic activity status, TQ dose, and plasma TQ concentration

A general linear model was developed to determine whether the combination of CYP2D6 metabolism status and TQ dose influenced an individual’s plasma TQ concentration. The model was significant (*P* < 0.001, *R*^2^ 68.5%); however, the only significant variable was TQ dose (*P* < 0.001). This suggests that after accounting for TQ dose, CYP2D6 metabolism status did not affect the plasma TQ concentration.

## DISCUSSION

This study aimed to investigate whether CYP2D6 affects plasma TQ concentration and anti-relapse efficacy of the new 8-aminoquinoline drug TQ. To achieve this goal, archived plasma samples from groups of Australian Defence Force (ADF) personnel deployed to PNG and Timor-Leste during 1999 and 2001 who participated in TQ presumptive anti-relapse therapy (PART, also known as terminal prophylaxis or postexposure prophylaxis) and prophylaxis studies were used to determine CYP2D6 genotype and deduced phenotype. Plasma TQ concentrations were available from earlier clinical studies and were used in this study to determine the association with CYP2D6 phenotypes. The participants deployed to PNG and Timor-Leste were pooled in the analysis for the same reasons outlined in our earlier CYP2D6 study of PQ (34).

The study initially consisted of 174 plasma samples stored since 1999; however, only 92 of these samples yielded good quality and quantity of genomic DNA allowing for a successful amplification of the entire CYP2D6 gene and subsequent sequencing. The remaining 47% of samples failed to amplify either the entire or some fragments of the gene. This was likely due to degradation of DNA over the 20-year plus storage period, despite the samples being stored at −80°C at the Australian Defence Force Malaria and Infectious Disease Institute (ADFMIDI). Furthermore, plasma samples are not an ideal source of genetic material compared to blood samples. Note that the plasma TQ concentrations measured in the early 2000s in samples collected from the 89 participants provide evidence of drug adherence in the PART (30) and chemoprophylactic (31, 33) studies of TQ.

For those participants from the relapse cohort, the shortest duration until relapse following TQ dosing was 67 days. Therefore, all relapses are considered true relapses of the previous *P. vivax* infection and not new infections since the relapse time exceeds four terminal elimination half-lives of TQ and the participants had returned to Australia prior to relapse. Our study also identified and included participants with CYP2D6 IM and PM, fulfilling the gap left by previous studies where no or a low number of subjects with PM status had been identified in the TQ arm (30, 31). This, combined with the measurement of plasma TQ concentrations in this study, provides the opportunity to detect possible interactions between CYP2D6 status and TQ anti-relapse efficacy.

This study provided several lines of evidence demonstrating that CYP2D6 was not a major contributor affecting plasma TQ concentrations and anti-relapse efficacy of TQ for radical cure and chemoprophylaxis. Firstly, the deduced CYP2D6 AS and metabolism status did not correlate with plasma TQ concentration. Instead, plasma TQ concentrations were shown to be directly dependent on the TQ dose regimen. Secondly, CYP2D6 genotype frequency, the deduced CYP2D6 AS, and metabolism status did not correlate with *P. vivax* relapses. Although the non-relapse group had a higher proportion of UM and NM and a lower proportion of IM and PM than the relapse group, the difference did not reach statistical significance.

These findings were reinforced by the linear modeling between participants’ TQ dosing regimens and plasma TQ concentrations against relapse outcomes, which showed a relationship between dosing regimens and plasma TQ concentrations, but not between CYP2D6 status and plasma TQ concentrations or between CYP2D6 status and relapse outcomes.

It is noteworthy that, although plasma TQ concentrations were dose-dependent in the PART studies (35), no statistical difference between the two dosing regimens and the risk of *P. vivax* relapses was found. This was unexpected as TQ’s anti-relapse efficacy has a clear dose-response relationship as shown by pooled individual data obtained from preregistration trials of TQ for radical cure (36). Watson et al. (36) using dose-response models showed that the FDA- and TGA-approved single-fixed 300-mg TQ adult dose combined with chloroquine (1,500-mg base given over 3 days) has suboptimal radical cure efficacy, with a mean recurrence proportion of 15.3% (95% CI: 9.6 to 21.7) at 4 months after follow-up. Increasing the fixed dose of TQ to 450 mg reduces the risk of *P. vivax* recurrence by more than two fold, with a mean recurrence proportion of 6.2% (95% CI: 3.2 to 11.5). Unlike the multicenter Phase 3 studies for radical cure conducted between 2010 and 2017 in 1,073 patients across nine countries (36), in the PART studies (30), the total TQ dose administered to the participants over three consecutive days of 600 mg and 1,200 mg was well above the recommended 450 mg fixed dose, with relapse rates of 5.0% and 6.3%, respectively. As there are no data available on the minimum inhibitory concentrations that prevent relapses of *P. vivax* infections and variability in hypnozoite susceptibility, the determination of an effective TQ dose for both radical cure and PART is still elusive.

Curiously, the study identified that participants with homologous CYP2D6 alleles had an increased risk of relapse compared to individuals with heterologous CYP2D6 alleles. This is mainly due to a higher risk of relapses in individuals who are predicted to have IM status with a homologous combination of alleles compared to those with a heterologous combination of alleles. In addition, all individuals with PM status resulted from a homologous combination of alleles. Nevertheless, the overall CYP2D6 metabolic status and AS were not associated with the risk of relapse. Another possibility could be due to possible unknown linkages of other genes to CYP2D6 that affect the anti-relapse efficacy of TQ. The relatively small sample size, particularly individuals with PM and IM status could also be a contributor. Further studies with larger sample sizes may provide a clearer answer.

As this was a retrospective study, not originally designed to investigate the relationship between CYP2D6 and TQ anti-relapse efficacy, the requirement to use plasma samples severely reduced the number of available participants due to poor DNA amplification for sequencing. This may have limited the statistical power of detecting a significant relationship. Future studies with larger sample sizes of blood samples from populations of diverse CYP2D6 activity status would be warranted.

In conclusion, unlike PQ where anti-relapse efficacy is affected by the CYP2D6 metabolic activity status, plasma TQ concentration and anti-relapse efficacy appear to be independent of CYP2D6 activity. While both PQ and TQ’s radical cure are restricted to patients with normal G6PD activity, TQ’s much longer half-life and non-reliance on CYP2D6 compared to PQ would enhance the adherence and coverage of TQ for radical cure and PART in settings where G6PD phenotypes can be readily screened.

## MATERIALS AND METHODS

### Study population

The participants were ADF personnel on peacekeeping duties in Bougainville, PNG, and in Timor-Leste who received TQ for PART (35), as well as chemoprophylaxis in Timor-Leste (37). The ADF personnel were administered one of three TQ regimens (400 mg per day for 3 days, 200 mg per day for 3 days, or 200 mg per day for 3 days followed by 200 mg per week). Despite TQ treatment, some participants experienced *P. vivax* relapses after returning to Australia. In the PART studies (30), the prevention relapse efficacy was 93.7% (355/379) for the participants treated with 400 mg TQ daily for 3 days and 95.0% (382/402) for 200 mg TQ daily for 3 days. For the participants on 200 mg TQ daily for 3 days followed by 200 mg TQ weekly for 6 months, the prevention relapse efficacy was 98.5% (455/462) (37, 38).

Plasma samples from participants who had experienced a *P. vivax* relapse upon return to Australia (relapse group) and a set of computer-generated randomized plasma samples from participants who did not experience a post-treatment *P. vivax* infection (non-relapse group) were selected for analysis in this study. All samples had been stored at −80°C. Participants’ *P. vivax* infection status was blinded to investigators until the laboratory work was completed, and the CYP2D6 activity score (AS)/metabolism status of all individuals had been determined in poor CYP2D6 metabolizers (AS ≤0.5) versus normal/ultrarapid metabolizers (AS >0.5) (39).

### Sample description

The clinical trials examined two different doses of TQ for PART with participants receiving either 400 mg or 200 mg once daily for 3 days (35). For the 3-day PART regimens (30), the participants were bled at about 12 hours after the last TQ dose. This time is close to the maximum plasma TQ concentrations in humans (39). For malaria chemoprophylaxis, the participants received a 3-day loading dose of 200 mg of TQ, followed by a weekly dose of 200 mg TQ for 6 months (31). Steady-state weekly TQ concentrations were achieved, with plasma samples collected at various times after the 4th, 8th, 16th, and 26th for pharmacokinetic sampling (33). For this study, the participant’s plasma samples were collected at various times after the fourth weekly dose, with a median blood collection time of 8.7 hours (range: 7.3–15.1).

Participants’ blood samples were centrifuged, and plasma was separated and stored in liquid nitrogen. The participants’ PART and prophylactic plasma samples were shipped to ADFMIDI (Brisbane, Australia) and Quintiles Limited (Edinburgh, United Kingdom), respectively, on dry ice. At ADFMIDI and Quintiles Limited, the samples were stored at −80°C and −70°C, respectively, until analysis.

### Plasma TQ concentrations

Plasma samples from participants in the PART studies (33, 35) and prophylactic study (31) were measured over 20 years ago, as part of previous studies, for TQ concentrations by high-performance liquid chromatography (32) at ADFMIDI and liquid chromatography-mass spectrometry (33) at Quintiles Limited.

### Amplification and sequencing of CYP2D6

Genomic DNA was extracted from the plasma samples and used to amplify and sequence human CYP2D6. DNA extraction, PCR amplification, Sanger sequencing, and allele naming were previously described by Chen et al. (34). CYP2D6 duplication and deletion primers were as described by Gressier et al. (40). Study design, study population, and sample processing procedures of this study are summarized in Fig. 1. A summary of SNP’s, insertions and deletions within the CYP2D6 sequences and their corresponding alleles, AS, and metabolic status, as well as relapse status of ADF personnel is provided in Table S3.

### CYP2D6 allelic type and activity score

CYP2D6 alleles were determined following the CYP2D6 naming convention outlined by the Pharmacogene Variation Consortium (https://www.pharmvar.org/gene/CYP2D6). A CYP2D6 AS was assigned for each allele, and the combination of AS defines the individual’s overall CYP2D6 activity and metabolism status as outlined in references (41), as shown in Table S2 in the supplemental material.

### Data analysis

All statistical analyses were performed using the statistical software package Jamovi (42). The analysis focused on (i) comparing the plasma TQ concentrations between dosing regimens (Kruskal-Wallis test) and relapse status (Mann-Whitney *U* test); (ii) comparing the distribution for days to relapse between TQ dosing regimens (Kruskal-Wallis test); (iii) testing the association between allele combination and relapse status (chi-square test) and CYP2D6 metabolism status and relapse (chi-square test); and (iv) investigating the effect of CYP2D6 metabolism status and TQ dosing regimens on plasma TQ concentrations in a general linear model.

## Data Availability

Data generated from this study are included in the published article or as supplementary information.

## SUPPLEMENTAL MATERIAL

Supplemental figure and tables aac.01014-23-s0001.docx Supplemental Tables S1, S2, Figure S1.

Supplemental table aac.01014-23-s0002.xlsx Supplemental Table S3. Summary of SNPs, Insertions and Deletions in CYP2D6 gene of ADF personnel (referenced to NCBI Reference Sequence NG_008374.4) and their corresponding alleles, AS and metabolic status as well as relapse status.

ASM does not own the copyrights to Supplemental Material that may be linked to, or accessed through, an article. The authors have granted ASM a non-exclusive, world-wide license to publish the Supplemental Material files. Please contact the corresponding author directly for reuse.

## References

[1] Mendis K, Sina BJ, Marchesini P, Carter R. 2001. The neglected burden of Plasmodium vivax malaria. Am J Trop Med Hyg 64:97–106. doi:10.4269/ajtmh.2001.64.97 11425182

[2] Price RN, Commons RJ, Battle KE, Thriemer K, Mendis K. 2020. Plasmodium vivax in the era of the shrinking P. falciparum map. Trends Parasitol 36:560–570. doi:10.1016/j.pt.2020.03.009 32407682 PMC7297627

[3] Twohig KA, Pfeffer DA, Baird JK, Price RN, Zimmerman PA, Hay SI, Gething PW, Battle KE, Howes RE. 2019. Growing evidence of Plasmodium vivax across malaria-endemic Africa. PLoS Negl Trop Dis 13:e0007140. doi:10.1371/journal.pntd.0007140 30703083 PMC6372205

[4] Price RN, Tjitra E, Guerra CA, Yeung S, White NJ, Anstey NM. 2007. Vivax malaria: neglected and not benign. Am J Trop Med Hyg 77:79–87. doi:10.4269/ajtmh.2007.77.79 18165478 PMC2653940

[5] Dini S, Douglas NM, Poespoprodjo JR, Kenangalem E, Sugiarto P, Plumb ID, Price RN, Simpson JA. 2020. The risk of morbidity and mortality following recurrent malaria in Papua, Indonesia: a retrospective cohort study. BMC Med 18:28. doi:10.1186/s12916-020-1497-0 32075649 PMC7031957

[6] White NJ. 2011. Determinants of relapse periodicity in Plasmodium vivax malaria. Malar J 10:297. doi:10.1186/1475-2875-10-297 21989376 PMC3228849

[7] Robinson LJ, Wampfler R, Betuela I, Karl S, White MT, Li Wai Suen CS, Hofmann NE, Kinboro B, Waltmann A, Brewster J, Lorry L, Tarongka N, Samol L, Silkey M, Bassat Q, Siba PM, Schofield L, Felger I, Mueller I. 2015. Strategies for understanding and reducing the Plasmodium vivax and Plasmodium ovale hypnozoite reservoir in Papua new guinean children: a randomised placebo-controlled trial and mathematical model. PLoS Med 12:e1001891. doi:10.1371/journal.pmed.1001891 26505753 PMC4624431

[8] Betuela I, Rosanas-Urgell A, Kiniboro B, Stanisic DI, Samol L, de Lazzari E, Del Portillo HA, Siba P, Alonso PL, Bassat Q, Mueller I. 2012. Relapses contribute significantly to the risk of Plasmodium vivax infection and disease in Papua New Guinean children 1-5 years of age. J Infect Dis 206:1771–1780. doi:10.1093/infdis/jis580 22966124

[9] Adekunle AI, Pinkevych M, McGready R, Luxemburger C, White LJ, Nosten F, Cromer D, Davenport MP. 2015. Modeling the dynamics of Plasmodium vivax infection and hypnozoite reactivation in vivo. PLoS Negl Trop Dis 9:e0003595. doi:10.1371/journal.pntd.0003595 25780913 PMC4364305

[10] Commons RJ, Simpson JA, Watson J, White NJ, Price RN. 2020. Estimating the proportion of Plasmodium vivax recurrences caused by relapse: a systematic review and meta-analysis. Am J Trop Med Hyg 103:1094–1099. doi:10.4269/ajtmh.20-0186 32524950 PMC7470578

[11] WHO. 2015. Guidelines for the treatment of malaria, 3rd Edn. World Health Organization, Geneva. http://www.who.int/malaria/publications/atoz/9789241549127/en 26020088

[12] Commons RJ, McCarthy JS, Price RN. 2020. Tafenoquine for the radical cure and prevention of malaria: the importance of testing for G6PD deficiency. Med J Aust 212:152–153. doi:10.5694/mja2.50474 32036613 PMC7064913

[13] Douglas NM, Poespoprodjo JR, Patriani D, Malloy MJ, Kenangalem E, Sugiarto P, Simpson JA, Soenarto Y, Anstey NM, Price RN. 2017. Unsupervised primaquine for the treatment of Plasmodium vivax malaria relapses in Southern Papua: a hospital-based cohort study. PLoS Med 14:e1002379. doi:10.1371/journal.pmed.1002379 28850568 PMC5574534

[14] Poespoprodjo JR, Burdam FH, Candrawati F, Ley B, Meagher N, Kenangalem E, Indrawanti R, Trianty L, Thriemer K, Price DJ, Simpson JA, Price RN. 2022. Supervised versus unsupervised primaquine radical cure for the treatment of falciparum and vivax malaria in Papua, Indonesia: a cluster-randomised, controlled, open-label superiority trial. Lancet Infect Dis 22:367–376. doi:10.1016/S1473-3099(21)00358-3 34710363 PMC8866132

[15] Brueckner RP, Lasseter KC, Lin ET, Schuster BG. 1998. First-time-in-humans safety and pharmacokinetics of WR 238605, a new antimalarial. Am J Trop Med Hyg 58:645–649. doi:10.4269/ajtmh.1998.58.645 9598455

[16] Kim YR, Kuh HJ, Kim MY, Kim YS, Chung WC, Kim SI, Kang MW. 2004. Pharmacokinetics of primaquine and carboxyprimaquine in Korean patients with vivax malaria. Arch Pharm Res 27:576–580. doi:10.1007/BF02980134 15202566

[17] Binh VQ, Chinh NT, Thanh NX, Cuong BT, Quang NN, Dai B, Travers T, Edstein MD. 2009. Sex affects the steady-state pharmacokinetics of primaquine but not doxycycline in healthy subjects. Am J Trop Med Hyg 81:747–753. doi:10.4269/ajtmh.2009.09-0214 19861604

[18] Dern RJ, Beutler E, Alving AS. 1954. The hemolytic effect of primaquine. II. The natural course of the hemolytic anemia and the mechanism of its self-limited character. J Lab Clin Med 44:171–176. doi:10.5555/uri:pii:0022214354902061 13184224

[19] Alving AS, Carson PE, Flanagan CL, Ickes CE. 1956. Enzymatic deficiency in primaquine-sensitive erythrocytes. Science 124:484–485. doi:10.1126/science.124.3220.484-a 13360274

[20] Ingelman-Sundberg M. 2004. Pharmacogenetics of cytochrome P450 and its applications in drug therapy: the past, present and future. Trends Pharmacol Sci 25:193–200. doi:10.1016/j.tips.2004.02.007 15063083

[21] Marcsisin SR, Reichard G, Pybus BS. 2016. Primaquine pharmacology in the context of CYP 2D6 pharmacogenomics: current state of the art. Pharmacol Ther 161:1–10. doi:10.1016/j.pharmthera.2016.03.011 27016470

[22] Sistonen J, Sajantila A, Lao O, Corander J, Barbujani G, Fuselli S. 2007. CYP2D6 worldwide genetic variation shows high frequency of altered activity variants and no continental structure. Pharmacogenet Genomics 17:93–101. doi:10.1097/01.fpc.0000239974.69464.f2 17301689

[23] Suarez-Kurtz G. 2021. Impact of CYP2D6 genetic variation on radical cure of Plasmodium vivax malaria. Clin Pharmacol Ther 110:595–598. doi:10.1002/cpt.2313 34042179

[24] Bennett JW, Pybus BS, Yadava A, Tosh D, Sousa JC, McCarthy WF, Deye G, Melendez V, Ockenhouse CF. 2013. Primaquine failure and cytochrome P-450 2D6 in Plasmodium vivax malaria. N Engl J Med 369:1381–1382. doi:10.1056/NEJMc1301936 24088113

[25] Pybus BS, Marcsisin SR, Jin X, Deye G, Sousa JC, Li Q, Caridha D, Zeng Q, Reichard GA, Ockenhouse C, Bennett J, Walker LA, Ohrt C, Melendez V. 2013. The metabolism of primaquine to its active metabolite is dependent on CYP 2D6. Malar J 12:212. doi:10.1186/1475-2875-12-212 23782898 PMC3689079

[26] Baird JK, Louisa M, Noviyanti R, Ekawati L, Elyazar I, Subekti D, Chand K, Gayatri A, Soebianto S, Crenna-Darusallam C, Djoko D, Hasto BD, Meriyenes D, Wesche D, Nelwan EJ, Sutanto I, Sudoyo H, Setiabudy R. 2018. Association of impaired cytochrome P450 2D6 activity genotype and phenotype with therapeutic efficacy of primaquine treatment for latent Plasmodium vivax malaria. JAMA Netw Open 1:e181449. doi:10.1001/jamanetworkopen.2018.1449 30646129 PMC6324265

[27] Vuong C, Xie LH, Potter BM, Zhang J, Zhang P, Duan D, Nolan CK, Sciotti RJ, Zottig VE, Nanayakkara NP, Tekwani BL, Walker LA, Smith PL, Paris RM, Read LT, Li Q, Pybus BS, Sousa JC, Reichard GA, Smith B, Marcsisin SR. 2015. Differential CYP 2D6 metabolism alters primaquine pharmacokinetics. Antimicrob Agents Chemother 59:2380–2387. doi:10.1128/AAC.00015-15 25645856 PMC4356838

[28] Marcsisin SR, Sousa JC, Reichard GA, Caridha D, Zeng Q, Roncal N, McNulty R, Careagabarja J, Sciotti RJ, Bennett JW, Zottig VE, Deye G, Li Q, Read L, Hickman M, Dhammika Nanayakkara NP, Walker LA, Smith B, Melendez V, Pybus BS. 2014. Tafenoquine and NPC-1161B require CYP 2D metabolism for anti-malarial activity: implications for the 8-aminoquinoline class of anti-malarial compounds. Malar J 13:2. doi:10.1186/1475-2875-13-2 24386891 PMC3893421

[29] Vuong C, Xie LH, Potter BM, Zhang J, Zhang P, Duan D, Nolan CK, Sciotti RJ, Zottig VE, Nanayakkara NP, Tekwani BL, Walker LA, Smith PL, Paris RM, Read LT, Li Q, Pybus BS, Sousa JC, Reichard GA, Smith B, Marcsisin SR. 2015. Differential cytochrome P450 2D metabolism alters tafenoquine pharmacokinetics. Antimicrob Agents Chemother 59:3864–3869. doi:10.1128/AAC.00343-15 25870069 PMC4468667

[30] St Jean PL, Xue Z, Carter N, Koh G, Duparc S, Taylor M, Beaumont C, Llanos-Cuentas A, Rueangweerayut R, Krudsood S, Green JA, Rubio JP. 2016. Tafenoquine treatment of Plasmodium vivax malaria: suggestive evidence that CYP2D6 reduced metabolism is not associated with relapse in the phase 2b DETECTIVE trial. Malar J 15:97. doi:10.1186/s12936-016-1145-5 26888075 PMC4757974

[31] Llanos-Cuentas A, Lacerda MVG, Hien TT, Vélez ID, Namaik-Larp C, Chu CS, Villegas MF, Val F, Monteiro WM, Brito MAM, Costa MRF, Chuquiyauri R, Casapía M, Nguyen CH, Aruachan S, Papwijitsil R, Nosten FH, Bancone G, Angus B, Duparc S, Craig G, Rousell VM, Jones SW, Hardaker E, Clover DD, Kendall L, Mohamed K, Koh G, Wilches VM, Breton JJ, Green JA. 2019. Single-dose tafenoquine to prevent relapse of Plasmodium vivax malaria. N Engl J Med 380:215–228. doi:10.1056/NEJMoa1710775 30650322 PMC6657226

[32] Kocisko DA, Walsh DS, Eamsila C, Edstein MD. 2000. Measurement of tafenoquine (WR 238605) in human plasma and venous and capillary blood by high-pressure liquid chromatography. Ther Drug Monit 22:184–189. doi:10.1097/00007691-200004000-00008 10774631

[33] Charles BG, Miller AK, Nasveld PE, Reid MG, Harris IE, Edstein MD. 2007. Population pharmacokinetics of tafenoquine during malaria prophylaxis in healthy subjects. Antimicrob Agents Chemother 51:2709–2715. doi:10.1128/AAC.01183-06 17517850 PMC1932489

[34] Chen N, Dowd S, Gatton ML, Auliff A, Edstein MD, Cheng Q. 2019. Cytochrome P450 2D6 profiles and their relationship with outcomes of primaquine anti-relapse therapy in Australian defence force personnel deployed to Papua New Guinea and East Timor. Malar J 18:140. doi:10.1186/s12936-019-2774-2 30999967 PMC6471761

[35] Elmes NJ, Nasveld PE, Kitchener SJ, Kocisko DA, Edstein MD. 2008. The efficacy and tolerability of three different regimens of tafenoquine versus primaquine for post-exposure prophylaxis of Plasmodium vivax malaria in the Southwest Pacific. Trans R Soc Trop Med Hyg 102:1095–1101. doi:10.1016/j.trstmh.2008.04.024 18541280

[36] Watson JA, Commons RJ, Tarning J, Simpson JA, Llanos Cuentas A, Lacerda MVG, Green JA, Koh G, Chu CS, Nosten FH, Price RN, Day NPJ, White NJ. 2022. The clinical pharmacology of tafenoquine in the radical cure of Plasmodium vivax malaria: an individual patient data meta-analysis. Elife 11:e83433. doi:10.7554/eLife.83433 36472067 PMC9725750

[37] Nasveld PE, Edstein MD, Reid M, Brennan L, Harris IE, Kitchener SJ, Leggat PA, Pickford P, Kerr C, Ohrt C, Prescott W, Tafenoquine Study Team. 2010. Randomized, double-blind study of the safety, tolerability, and efficacy of tafenoquine versus mefloquine for malaria prophylaxis in nonimmune subjects. Antimicrob Agents Chemother 54:792–798. doi:10.1128/AAC.00354-09 19995933 PMC2812156

[38] Dow GS, McCarthy WF, Reid M, Smith B, Tang D, Shanks GD. 2014. A retrospective analysis of the protective efficacy of tafenoquine and mefloquine as prophylactic anti-malarials in non-immune individuals during deployment to a malaria-endemic area. Malar J 13:49. doi:10.1186/1475-2875-13-49 24502679 PMC3942710

[39] Brueckner RP, Lasseter KC, Lin ET, Schuster BG. 1998. First-time-in-humans safety and pharmacokinetics of WR 238605, a new antimalarial. Am J Trop Med Hyg 58:645–649. doi:10.4269/ajtmh.1998.58.645 9598455

[40] Gressier F, Verstuyft C, Hardy P, Becquemont L, Corruble E. 2015. Response to CYP2D6 substrate antidepressants is predicted by a CYP2D6 composite phenotype based on genotype and comedications with CYP2D6 inhibitors. J Neural Transm (Vienna) 122:35–42. doi:10.1007/s00702-014-1273-4 25047911

[41] Caudle KE, Sangkuhl K, Whirl-Carrillo M, Swen JJ, Haidar CE, Klein TE, Gammal RS, Relling MV, Scott SA, Hertz DL, Guchelaar HJ, Gaedigk A. 2020. Standardizing CYP2D6 genotype to phenotype translation: consensus recommendations from the clinical pharmacogenetics implementation consortium and Dutch pharmacogenetics working group. Clin Transl Sci 13:116–124. doi:10.1111/cts.12692 31647186 PMC6951851

[42] Laing AB. 1965. Treatment of acute falciparum malaria with sulphorthodimethoxine (fanasil). Br Med J 1:905–907. doi:10.1136/bmj.1.5439.905 14257406 PMC2165621

