# Supplementary material for: Cytochrome P450 2D6 profiles and anti-relapse efficacy of tafenoquine against Plasmodium vivax in Australian Defence Force personnel
Source: Antimicrob Agents Chemother. 2023 Nov 16;67(12):e01014-23. doi: 10.1128/aac.01014-23 (PMC10720419; doi:10.1128/aac.01014-23)
Supplement: Supplemental figure and tables — Supplemental Tables S1, S2, Figure S1. [file aac.01014-23-s0001.docx]

# Supplemental MATERIAL

#

**TABLE S2** Combinations of two CYP2D6 alleles’ functionality and corresponding activity score (AS) to predict CYP2D6 metabolism status for an individual.

| Allele functionality  /AS | | Allele 2 | | | |
| --- | --- | --- | --- | --- | --- |
|  |  | IF / 2 | FF / 1 | RF / 0.25 -0.5 | NF / 0 |
| Allele 1 | IF / 2 | UM / 4 | UM / 3 | UM / 2.25 - 2.5 | UM / 2 |
|  | FF / 1 | UM / 3 | NM / 2 | NM / 1.25 - 1.5 | NM /1 |
|  | RF / 0.25 - 0.5 | UM 2.25 -2.5 | NM / 1.25 - 1.5 | IM / 0.5 - 1 | IM / 0.25 - 0.5 |
|  | NF / 0 | UM / 2 | NM / 1 | IM / 0.25 - 0.5 | PM / 0 |

Increased function: IF; Fully functional: FF; Reduced function: RF; No function: NF


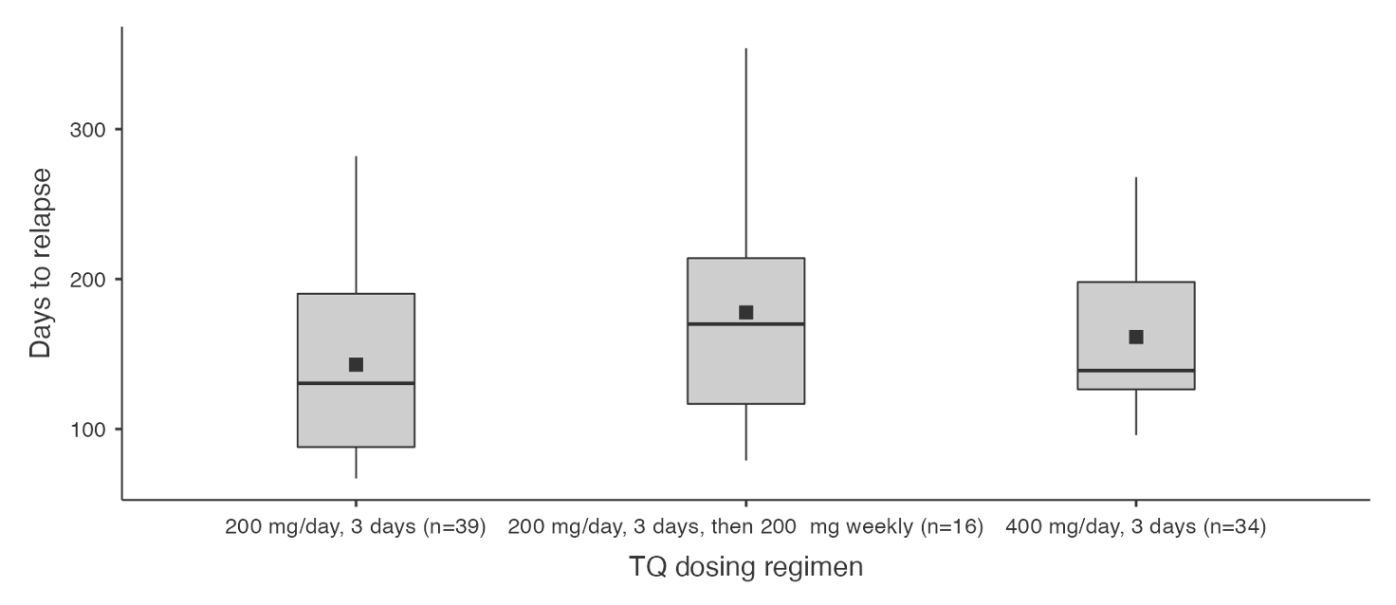


# FIG S1 TQ dosing regimen and days from final TQ dose until relapse. Mean and 95% confidence intervals are represented as horizontal lines.
